# Educational achievement and bullying: The mediating role of psychological difficulties

**DOI:** 10.1111/bjep.12511

**Published:** 2022-05-21

**Authors:** Daráine Murphy, Sophie J. Leonard, Laura K. Taylor, Flavia H. Santos

**Affiliations:** ^1^ 8797 University College Dublin Dublin 4 Ireland

**Keywords:** bullying, growing up in Ireland, literacy, mediation analysis, numeracy, lliteracy

## Abstract

**Background:**

Bullying has a profound and enduring impact on academic achievement. However, there is a lack of clarity surrounding the specific mechanisms of this relationship.

**Aims:**

This study examined the link between bullying at age 9 and Numeracy/Literacy achievement at age 15 to determine if this relationship is partially or fully explained by psychological difficulties at age 13.

**Sample:**

Secondary data analysis was completed on waves 1, 2 and 3 of child cohort (Cohort’98) of the Growing Up in Ireland (GUI) study, respectively, at 9 years (*N *= 8568), 13 years (*N* = 7527) and 15 years of age (*N *= 6216).

**Results:**

Longitudinal path mediation model was conducted with bullying at age 9 as the predictor, total (emotional and behavioural) difficulties at age 13 as the mediator and Numeracy/Literacy scores at age 15 as outcomes revealing significant indirect effects of bullying on achievement, via psychological difficulties.

**Conclusions:**

We discuss the impact of bullying on the student's psychological well‐being, the relationship between bullying and academic attainment and how this may be tackled to avoid consequences throughout education and later in life.

**Educational Impact and Implications:**

This study emphasizes the need for schools to address the emotional and behavioural difficulties occurring as a result of bullying in order to improve the overall educational experience of a child. Existing interventions can be built upon by focusing on the continuous remediation of such psychological difficulties.

## BACKGROUND

Bullying in schools is a pervasive problem with profound implications for both the students and the school environment (Gaffney et al., 2021; Huang, 2020). One of the most used definitions of bullying was developed by Olweus (1993), who stated that bullying occurs when a student is subjected to aggressive behaviour repeatedly by another over a period. It extends beyond teasing in the sense that it often involves a distinct social or physical imbalance of power between the intimidator and the victim (Centre for Disease Control & Prevention, 2014). A study by the Health Behaviour in School‐aged Children found that 25% of adolescents in Ireland reported being bullied by a classmate in 2014 (Gavin et al., 2015). Overall, bullying tends to decrease with age up until the end of second level education, although the ages of 8–13 are salient for such behaviours (Nansel et al., 2001; Pellegrini, & Bartini, 2001).

Unsurprisingly, bullying has been found to have a negative impact on the victim's well‐being, regardless of the type of bullying, because both the victimization and marginalization can leave the child distressed and in turn resenting their school experience (Schwartz et al., 2005). While factors such as self‐esteem and resilience can buffer the negative effects of bullying (Narayanan, & Betts, 2014), bullying is still associated with a variety of internalizing symptoms including, sleep difficulties (Donoghue, & Meltzer, 2018), anxiety (Dantchev et al., 2019), depression (Dantchev et al., 2019), suicidal ideation (Kim et al., 2005), psychotic‐like symptoms (Campbell & Morrison, 2007; Catone et al., 2017), loneliness, isolation (Cao et al., 2020) and somatic symptoms (Espejo‐Siles et al., 2020). Furthermore, bullying may put children at risk for the development of behavioural problems including aggression and delinquency (Hanish & Guerra, 2002).

The psychological impacts of bullying may even extend beyond school and last throughout adulthood (Brimblecombe et al., 2018). Data from the 1958 British Cohort Study found that individuals who were bullied in childhood had higher levels of psychological distress, a lack of social relationships, in addition to fewer employment opportunities and less accumulated wealth at the age of 50 (Brimblecombe et al., 2018; Takizawa et al., 2014). From a policy perspective, the impact of bullying on educational attainment and long‐term employment are a cause for concern.

Numeracy and literacy comprise two of the most essential facets of education. From early childhood precedence is given within the schooling system to developing attainment in mathematics and literacy, and levels of educational performance in these faculties have been found to equate to various outcomes later in life (Duncan et al., 2007; Watts et al., 2014). Studies show that mathematical and literacy attainment at the age of 7 is a strong predictor of socio‐economic status by the age of 42 as well as academic motivation and duration of education, with these relationships extending beyond factors such as general intelligence or socio‐economic status at birth (Ritchie & Bates, 2013). Though the processes underlying learning and performance in mathematics are generally treated separately from those of literacy (Carreiras et al., 2015; Zoccolotti et al., 2021), research shows that effects of bullying can still be inhibitory to both. Looking at longitudinal evidence, Smith and Skrbiš (2016) analysed data from the Social Futures and Life Pathways (‘Our Lives’) project and found that bullying was negatively associated with educational attainment when leaving school. Individuals who were persistently bullied performed 12% worse on their final exams compared to individuals who had never been bullied. Fry et al. (2018) systematically reviewed the literature and found that bullying experienced in childhood is related to increased levels of dropout, absenteeism (especially for males) and poorer achievement on standardized test scores. Similarly, Ladd et al. (2017) followed children from kindergarten to grade 12 and showed that any form of peer victimization disrupts children's academic achievement particularly during the earlier or foundation years, but this is more so for mathematics achievement and the impact on literacy scores was not as extreme.

Current knowledge on the relationship between academic performance and bullying is underpinned by two prevailing arguments in the literature. Some researchers propose that victimized students yield poorer academic performance because they feel unhappy in school and in turn begin to disengage, thus reducing their classroom performance and school attendance (Li et al., 2020). Other researchers have proposed that bullying impacts on academic achievement because it can cause the student considerable distress, which in turn influences performance (Schwartz et al., 2005). Graham et al. (2003) found that rather than the act of bullying having an impact on academic performance, instead perceiving one‐self as a victim undermines school performance due to psychological costs, such as depression and anxiety. As such, emotional and behavioural facets of development must not be overlooked when assessing the impact of bullying on long‐term educational outcomes. Previous longitudinal studies indicate these aspects of child psychological health indeed serve to either bolster or impede academic performance throughout education (Hawkins et al., 2008; Jones et al., 2011). Recent research posits that the externalized behavioural difficulties in particular, as measured by the SDQ, hampered both mathematics and literacy attainment in a sample of 5–13 year olds (Kirkøen et al., 2021).

Indications from previous literature suggest the relationship between childhood total (emotional and behavioural) difficulties and mathematics education is a seemingly reciprocal one. Dobbs et al. (2006) found that attentional problems and social withdrawal were indicative of lower mathematical ability while salient emotional skills such as initiative‐taking and self‐control indicated increased skill in mathematics. Furthermore, implementation of mathematics intervention in preschool worked to improve total difficulties in the cohort, improving psychological health. To extend to literacy attainment, Oberle et al. (2014) found that teacher‐reported emotional competence in sixth grade was longitudinally predictive of both numeracy and literacy scores. On the contrary, findings from the Canadian National Longitudinal Survey of Children and Youth suggest that parent‐reported emotional and behavioural difficulties in kindergarten did not act as a predictor of academic attainment in third grade (Beran et al., 2008; Duncan et al., 2007; Romano et al., 2010). This calls into question the true nature of the role of total difficulties in academic achievement throughout education and how this might interact with incidences of bullying.

Various further factors may impact the predicted relationship between bullying and educational attainment, and it remains necessary to be mindful of these factors when testing this relationship. Gender is particularly important to examine considering ever‐growing evidence demonstrating an absence of difference in early mathematics achievement despite contrasting societal stereotypes (Tomasetto et al., 2011). This may explain higher tendencies towards mathematics anxiety in school‐aged girls than their male counterparts (Szczygiel, 2021), while gender differences in verbal anxiety are not so pronounced (Lauer et al., 2018). Socioeconomic factors such as mother's education have also been shown to influence achievement in literacy and numeracy (Blums et al., 2017; Chiu & Chow, 2015). Along with income and social class, this has been classified as a predictor of bullying in school (Elgar et al., 2009; Pitsia & Mazzone, 2020) as well as determinants of long‐term implications of bullying (Due et al., 2009). Adding to this, children's individual attitudes towards their education and views of self also contribute to educational attainment, becoming particularly relevant in the presence of bullying (Ladd et al., 2017).

There is a need for further research to add more clarity to the relationship between both numeracy and literacy attainment, and bullying (Nakamoto & Schwartz, 2010). While we know there is a strong relationship between bullying and educational outcomes there is a lack of comprehensive data on this relationship (Fry et al., 2018). Studies that have previously explored this relationship have also employed a short‐term follow up (1 year or less), had a small sample size (less than 1 thousand participants) and have failed to control for key covariates including attitudes towards school and socioeconomic status (Juvonen et al., 2000; Rueger & Jenkins, 2014). Therefore, the aim of the study was to address the limitations of the previous literature and determine the extent to which total psychological difficulties at age 13 mediates the relationship between bullying at age 9 and Numeracy and Literacy attainment at age 15 using a large‐scale longitudinal cohort study, Growing Up in Ireland. Our hypotheses are that total difficulties at age 13 will fully mediate the relationship between bullying at the age of 9 and both mathematical and literacy attainment at age 15.

## METHODS

### Growing up in Ireland

Growing Up in Ireland is the national longitudinal study of children in Ireland. In 2006, there were approximately 56,500 nine‐year olds registered in the Census of the population. To obtain a representative cohort, a sample of 8500 nine‐year‐old children was selected, which would represent 14% of the population or one in every seven children (Murray et al., 2010). The sample design for the cohort was based on a two‐stage selection process in which the school was the primary sampling unit and the children within the school were the secondary sampling unit. In the first stage of sampling, 1105 primary schools from the national total of 3177 were selected using a probability proportionate to size (PPS) sampling method. In the second stage, a random sample of eligible children was selected within each school. At the school level, a response rate of 82% was achieved yielding a sample of 910 primary schools, while at the level of the household (i.e., eligible child selected within the school), a total of 57% of children and their families participated in the study yielding a final sample of 8568 children (Murray et al., 2010). Data collection for the second wave at 13 years took place between August 2011 and March 2012 and resulted in responses from 7525 young people and their families, yielding a response rate of 89% (Quail et al., 2014). Data collection for the third wave at 17 took place between August 2015 and August 2016 and resulted in responses from 6216 cases, yielding a response rate of 74% (Murphy et al., 2019).

#### Study design and sample

The data used in this study were collected as part of the child cohort (Cohort’98) of the Growing Up in Ireland (GUI) study who participated in waves 1, 2 and 3 of the study, respectively, at 9 years (*N *= 8568), 13 years (*N* = 7527) and 17 years of age (*N* = 6216). Only 9‐year‐olds who responded to the question on bullying were included in the analysis (*N *= 8214).

#### Data collection procedures

Trained social interviewers conducted interviews with the study child and their primary caregiver (and secondary caregiver where applicable) in the home. The primary caregiver was nominated by the family as the person who provides the most care and is most knowledgeable about the study child, in 98% of cases this was the child's biological mother (Quail et al., 2014). The main interviews were completed on a Computer Assisted Personal Interview (CAPI) basis and there was also a self‐complete paper‐based supplement that contained some potentially sensitive questions. Ethics for this study was granted by University College Dublin Research Ethics Committee (HS‐E‐19‐14‐Santos).

### Measures

#### Independent variables

##### Bullying

The question on bullying was developed by the *Growing Up in Ireland* study team and the prevalence of bullying experienced by the study child in the past year was explored. The question asked was ‘Thinking back over the last year would you say that anyone (either a child or adult) picked on you?’. The questions were answered yes/no by the study child.

#### Mediator

The Strengths and Difficulties Questionnaire is a measure of psychological health in terms of emotional and behavioural difficulties at a stable rate across development (Sosu & Schmidt, 2017). It contains 25 items, whereby the respondent must indicate their level of agreement with each item on a three‐point scale of ‘Certainly true’, ‘Somewhat true’ or ‘Not true’. Item scores vary from 0 to 2 depending on the type of endorsement, and the total difficulties score ranges from 0 to 40. The scale contains five subscales: hyperactivity, emotional symptoms, conduct problems, peer relationship problems and pro‐social behaviour. The total difficulties score was calculated by summing the four deficit‐focused scales (i.e., all except the prosocial behaviour scale; Quail et al., 2014). While the overall scale assesses total psychological difficulties, the emotional and peer relationship subscales represent internalized symptoms, with the conduct and hyperactivity subscales representing externalized symptoms (Goodman, 2001; Kirkøen et al., 2021). The scale has previously been found to have good internal reliability (α = .73), construct validity and discriminant validity (Goodman, 2001; Hawes & Dadds, 2004; Murray et al., 2010). The SDQ score measured at age 9 was included as a control and at age 13 as the mediator. It is important to note as all exogenous variables at age 9 were allowed to correlate. Any potential association between the SDQ‐Peer Relations subscale and the bullying item were estimated by the model. Therefore, the direct path to later SDQ Total Problems is what is ‘left over’ after accounting for this earlier association.

#### Dependent variables

##### Numeracy and literacy achievement scores

Mathematics and literacy achievement were measured based on the young person's Junior Certificate results, as reported by the young person at 17 years. It is important to note that while the results were reported at age 17, the study child would have taken the exam at the end of their third year in secondary school (on average age 15 years) in Ireland. The exam is based on the curriculum for the subject. The exam can be taken at ordinary or higher level, mathematics can also be studied at foundation level. For this study, a composite score was created based on the student's grade in the subject; this was based on a previous composite score used by the Growing Up in Ireland study team (McNamara et al., 2018). The lowest score (0) indicates a failure at all levels, while higher scores (10,9,8) indicate higher scores at the highest level (see Table 1).

**TABLE 1:** Calculation of mathematics & literacy composite score

Grade	Higher level	Ordinary level	Foundation level
A	10	7	4
B	9	6	3
C	8	5	2
D	7	4	1
E, F, G	0	0	0

Note

Ref: McNamara et al. (2018).

#### Covariates

##### Numeracy and literacy achievement at age 9

The Drumcondra Maths and Reading Tests are developed for Irish school children and are linked to the national curriculum (Educational Research Centre, 2006). The tests are grade‐specific and are strongly linked to the syllabus for each year. The version used in Growing Up in Ireland was designed specifically for the study.

##### Gender

9‐year‐olds gender (Male/Female) as reported by the Primary Caregiver.

##### Income

Income was self‐reported by the Primary Caregiver when the child was age 9. Disposable household income is recorded as total gross household income less statutory deductions of income tax and social insurance contributions. Household equivalized income is calculated as disposable household income divided by equivalized household size. This gives a measure of household disposable income which has been ‘equivalised’ to account for the differences in size and composition of households in terms of the number of adults and/or children they contain. This was then divided into quintiles.

##### Social class

Social Class of Primary and Secondary Caregiver is derived from their occupation. Both caregivers (where relevant) of the 9‐year‐old were asked to provide details on their occupation, from current, or previous employment outside the home. This was then used to generate a social class classification for both Primary and Secondary Caregiver. These included professional/managerial (doctors, teachers, engineers), intermediate (hairdressers, drivers, bookkeepers), semi‐skilled/unskilled (care assistants, waiter/waitress, cleaners) and unemployed (those that have never worked).

##### Mothers education

The Primary Caregiver was asked to rate their highest level of educational attainment from ‘Primary or less’ to ‘Postgraduate level of education’ when the child was aged 9. Level of education was then recoded by the research team into a four‐fold classification system: Primary Level, Secondary Level, Diploma/Certificate and Degree.

##### Child's attitudes towards school at age 9

The child was asked how they feel about school on a three‐point scale from ‘always like it’ = 1 to ‘never like it’ = 3.

##### Child's perception of performance in school at age 9

The child was asked how well they were doing in their school work on a three‐point scale from ‘well’ = 1 to ‘poorly’ = 3.

##### Child's attitudes towards mathematics at age 9

The child was asked if they find maths difficult on a three‐point scale from ‘always like it’ = 1 to ‘never like it’ = 3.

##### Child's attitudes towards reading at age 9

The child was asked if they like reading on a three‐point scale from ‘always like it’ = 1 to ‘never like it’ = 3.

### Data analytic plan

Each wave of the GUI data was merged, and preliminary analysis was conducted using SPSS version 24. All data analyses were conducted in Mplus 7 (Muthén & Muthén, 2004) with full‐information maximum likelihood, which estimates unbiased coefficients for data that are missing at random (Enders, 2010). The longitudinal, path mediation model was estimated with bias corrected bootstrapped indirect effects with 1000 replications. The model was fully identified and therefore, fit statistics were not estimated. All endogenous variables were allowed to be correlated, and the error variances for all endogenous outcomes were estimated.

## RESULTS

### Missing data analysis

Attrition analysis was conducted to determine if there were differences in key variables based on those who remained in the study versus those who dropped out at 13 years, 17 years and who dropped out at 13 but returned at age 17. For psychological difficulties, difference was noted for those who remained at all 3 waves (*M *= 7.19, *SD* = 4.94) and those who dropped out at age 17 (*M *= 7.80, *SD* = 5.29). For the child's literacy scores at age 9, difference was noted for those who remained at all 3 waves (*M *= .334, *SD* = 0.10) and those who dropped out at each wave (9 only *M* = 0.299 *SD* = 0.086, 9 and 17 only *M* = 0.299 *SD* = 0.083 and 9 and 13 only *M* = 0.310 *SD* = 0.095). Similarly for the child's mathematics scores at age 9, difference was noted for those who remained at all 3 waves (*M* = 0.383 *SD* = 0.55) and those who dropped out at each wave (9 only *M* = 0.374 *SD* = 0.044, 9 and 17 only *M* = 0.372, *SD* = 0.041 and 9 and 13 only *M* = 0.378 *SD* = 0.052). Income differences were noted between those who remained at all 3 waves (*M *= 3.41, *SD* = 1.34) and those who dropped out at each wave (9 only *M *= 2.99 *SD* = 1.45, 9 and 17 only *M *= 3.06 *SD* = 1.38 and 9 and 13 only *M* = 3.19 *SD* = 1.42). For mother's education differences were noted between those who remained at all 3 waves (*M* = 3.77, *SD* = 1.25) and those who dropped out at each wave (9 only *M* = 3.33 *SD* = 1.28, 9 and 17 only *M* = 3.30 *SD* = 1.32 and 9 and 13 only *M* = 3.43 *SD* = 1.28).

### Preliminary analysis

Of the sample of 9‐year‐olds 51.6% were female. The majority had positive attitudes towards school (94.4%), however, differences were noted in relation to if the child was bullied (*M* = 1.84 *SD* = 0.009) or not (*M* = 1.77; *SD* = 0.007) *t*(6819.13) = 6.02, *p *< .05, with children who reported being bullied having poorer attitudes towards school. Similar trends were noted with regards to how the child felt they were doing in their schoolwork. The majority felt they were doing well in their schoolwork (63.7%). However, differences were noted in relation to if the child was bullied (*M *= 1.39; *SD* = 0.009) or not (*M *= 1.35; *SD* = 0.007) *t*(6482.29) = 3.45, *p *< .05, with children who had been bullied reporting they were not doing as well. In relation to their attitudes towards numeracy and literacy, 46.6% reported always liking mathematics and 59.7% reporting that they always liked literacy at age 9. No differences were noted between those who reported they were bullied or not in relation to attitudes towards literacy. Those who had been bullied were significantly more likely to have poorer attitudes towards mathematics (Reported bullying: *M *= 1.66; *SD* = 0.012, No bullying: *M *= 1.61; *SD* = 0.009) *t*(6279.96) = 3.24, *p *< .05) See Table 2 for detailed demographic information.

**TABLE 2:** Demographic characteristics of the sample (*N* = 8214)

Family demographics	*N*	%
Mother's education		
Primary level education	1431	17.4%
Secondary level education	2592	31.6%
Certificate/diploma	2040	24.8%
Degree	2151	26.2%
Income (quintiles)		
Income 1 (lowest)	1005	13.2%
Income 2	1314	17.2%
Income 3	1520	19.9%
Income 4	1733	22.7%
Income 5 (highest)	2048	26.9%
Child demographics		
Gender		
Female	4242	51.6%
Male	3972	48.4%
Child likes school		
Always like it	2108	25.7%
Sometimes like it	5628	68.7%
Never like it	456	5.6%
How child does in school		
Well	5234	63.9%
Average/Ok	2891	35.2%
Poorly	60	.7%
Child like Maths		
Always like it	3829	46.7%
Sometimes like it	3581	43.7%
Never like it	781	9.5%
Child like reading		
Always like it	4901	59.8%
Sometimes like it	2955	36.1%
Never like it	336	4.1%

Table 3 reports the means, standard deviations, ranges and bivariate correlations for the demographic controls and primary study variables. Overall, the model was a good fit to the data, *N* = 8214, χ^2^(10) = 297.49, *p* < .05; CFI = .97; TLI = .89; SRMR = .02; RMSEA = .06 (CI: 0.05, 0.07). Regarding the demographic and auto‐regressive controls, final mathematics achievement scores were significantly predicted by all except child gender and how much they liked school or reported doing well in school. That is, mother's education (β = .15, *p* < .001), income quantile (β=.12, *p* <.001), liking math at age 9 (β=.05, *p* <.001), and both literacy (β = .20, *p* < .001) and math (β = .27, *p* < .001) scores at age 9 were all positively linked to later math achievement, whereas psychological difficulties at age 9 (β = −.06, *p* < .001) and liking reading at age 9 (β = −.03, *p* < .01), were negatively related to later mathematics achievement. Final literacy achievement scores were positively predicted by mother's education (β = .09, *p* < .001), income quantile (β = .11, *p* < .001), both literacy (β = .28, *p* < .001) and mathematics (β = .12, *p* < .001) scores at age 9, liking reading at age 9 (β = .03, *p* < .05) and self‐report of doing well in school at age 9 (β = .04, *p* < .01), while scores were negatively related to earlier psychological difficulties (β = −.06, *p* < .01). Child gender was also related to final scores; girls had higher literacy scores than boys (β = .27, *p* < .001). In summary, with a few small differences, the pattern of findings for the demographic controls was largely similar for Numeracy and Literacy achievement.

**TABLE 3:** Means, standard deviations, ranges and bivariate correlations for the demographic controls and primary study variables

	*M*	*SD*	1	2	3	4	5	6	7	8	9	10	11	12	13	14
1. Bullying at age 9	38.6% Bullied (1) 38.6% Not (0)			−.025*	−.082**	−.017	−.041***	−.068***	.178***	.143***	−.028*	−.016	.066***	.039***	.037**	.019
2. Female	51.6% Female (1) 48.4% Male (0)		−.025*		−.011	−.108***	−.026*	.121***	−.049***	−.046***	−.025*	−.039***	−.170***	−.030**	.064***	−.116***
3. Maths Achievement at age 17	0	1	−.082***	−.011		.271***	.432***	.568***	−.309***	−.325***	.300***	.335***	−.022	−.099***	−.077***	−.035**
4. Maths Achievement at age 9	0	1	−.017	−.108***	.271***		.377***	.164***	−.092***	−.073***	.093***	.109***	.001	−.080***	−.081***	−.002
5. Literacy Achievement at age 9	0	1	−.041***	−.026*	.423***	.377***		.407***	−.222***	−.202***	.215***	.261***	−.022*	−.103***	.017	−.187***
6. Literacy Achievement at age 17	0	1	−.068**	.121***	.568***	.164***	.407***		−.274***	−.283***	.254***	.260***	−.055***	−.108***	.003	−.122***
7. SDQ at age 9	7.354	5.012	.178***	−.049***	−.309***	−.092***	−.222***	−.274***		.627***	−.154***	−.190***	.086***	.118***	.070***	.061***
8. SDQ at age 13	6.470	4.999	.143***	−.046***	−.325***	−.073***	−.202***	−.283***	.627***		−.143***	−.165***	.059***	.083***	.060***	.054***
9. Income	3.329	1.376	−.028*	−.025*	.300***	.093***	.215***	.254***	−.154***	−.143***		.407***	.038**	−.035**	.027*	−.029*
10. Mothers Education	3.663	1.274	−.016	−.039***	.335***	.109***	.261***	.260***	−.190***	−.165***	.407***		.024*	−.023*	.022*	−.044***
11. Child likes school	1.80	.522	.066***	−.170***	−.022	.001	−.022*	−.055***	−.086***	.059***	.038**	.024*		.154***	.227***	.220***
12. Child self‐reported performance in school	1.37	.497	.039***	−.030**	−.099***	−.080***	−.103***	−.108***	.118***	.083***	−.035**	−.023*	.154***		.152***	.125***
13. Attitudes towards Maths at age 9	1.63	.651	.037**	.064***	−.077***	−.081***	.017	.003	.070***	.060***	.027*	.022*	.227***	.152***		−.028*
14. Attitudes towards Literacy at age 9	1.44	.573	.019	−.116***	−.035**	−.002	−.187***	−.122***	.061***	.054***	−.029*	−.044***	.220***	.125***	−.028*	

^*^ *p* < .05, ^**^ *p* < .01, ^***^ *p* < .001.

Regarding the longitudinal mediational paths of interest, first, bullying at age 9 predicted greater psychological difficulties at age 13 (β = .02, *p* < .01). More specifically, children who reported being bullied also reported lower psychological problems compared to children who were not bullied. Second, psychological difficulties at age 13 predicted lower mathematics achievement (β = −.15, *p* < .001) and Literacy achievement (β = −.13, *p* < .001) at age 15. In other words, greater psychological difficulties were related to lower math and literacy scores.

**FIGURE 1:**
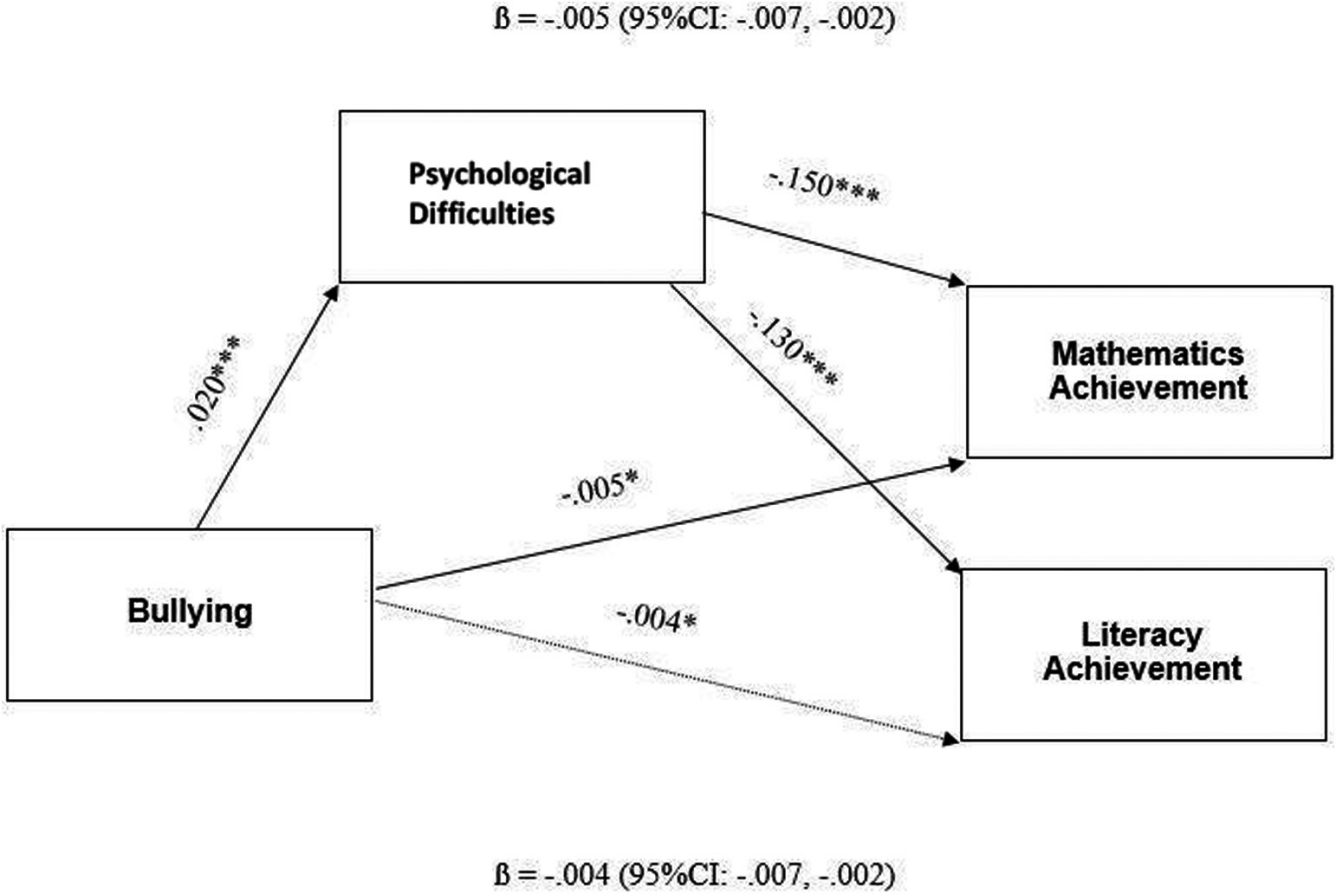
Bootstrapped mediation model of the indirect effect of bullying at age 9 on Literacy and Mathematics Achievement at age 15, via psychological difficulties at age 13 (*N* = 8214). Endogenous variables allowed to correlate. Standardized regression coefficients reported. Non‐significant paths indicated with dotted lines and indirect effects depicted with a dashed line. *p < .05, ***p < .01

Finally, the indirect effects for mathematics (β = −.005 [95% CI: −0.007, −0.002]) and literacy (β = −.004 [95% CI: −0.007, −0.002]) achievement were both significant. Indicating partial mediation for mathematics achievement, there remained a direct effect of bullying at age 9 (β = −.02, *p* < .05); those who reported being bullied scored lower on their mathematics junior cert than those who did not report being bullied. The direct effect from bullying to later Literacy scores was non‐significant. The final model accounted for 39% of the variance in numeracy achievement and 31% of literacy achievement. In summary, controlling for a number of important demographic variables as well as earlier psychological difficulties and performance in these academic domains, the effect of bullying on later numeracy and literacy achievement was mediated by psychological difficulties over time (Figure 1).

## DISCUSSION

This study explored the extent to which psychological difficulties mediate the relationship between bullying and both Numeracy and Literacy attainment. The results show that when controlling for a number of demographic and educational variables, psychological difficulties partially mediated the relationship between bullying at age 9 and both Numeracy and Literacy attainment later in education. The occurrence of bullying at age 9 predicted higher levels of psychological difficulties at age 13 and ultimately lower Junior Certificate scores for Mathematics and Literacy at age 15 for children who reported being bullied when compared with those who did not.

These findings echo those of previous research showing psychological adjustment to mediate the relationship between bullying (verbal, physical and relational) and academic achievement. The study also used a longitudinal design, however, it was short term from fall to spring in an academic year (Rueger & Jenkins, 2014). Similarly, Juvonen et al. (2000) found that bullying predicted psychological maladjustment, which in turn was related to poorer school adjustment. They did not find that psychological adjustment mediated the relationship between bullying and school achievement, however, their follow‐up period was only one year. Our findings further support the results of these studies by demonstrating that bullying can indirectly impact academic achievement via psychological difficulties over a four‐year period. This would indicate that it is the child's response to the bullying situation which has more of an impact than the bullying experience.

This model accounted for 39% of the variance in Numeracy attainment scores in comparison to 31% of the variance in Literacy attainment scores. Children who had been bullied reported significantly lower levels of interest in mathematics in comparison to those who had not been bullied, similarly children who had been bullied reported finding mathematics more difficult in comparison to those who had not been bullied. Such differences were not reported in relation to interest and difficulty in Literacy. Ladd et al. (2017) have previously shown that bullying tends to have a more profound impact on mathematics achievement in comparison to literacy scores. In many cases, students already have more negative attitudes and emotions towards mathematics and these negative experiences are associated with anxiety, shame, inadequacy, anxiety and hopelessness (Frenzel et al., 2007; Larkin & Jorgensen, 2016). In recent years, in particular, mathematics anxiety has been established in longitudinal research as a key predictor of mathematics achievement across education and development (Cargnelutti et al., 2017), though the exact cognitive processes through which this occurs are still unclear (Szczygiel, 2021). If students are experiencing the emotional impact of bullying coupled with already inherently negative feelings towards their mathematical ability, a more pronounced impact on their mathematical achievement may be expected (Stankov et al., 2014).

No gender differences were observed in the data in terms of Mathematics performance. This is reminiscent of several other studies in the field (Lachance & Mazzocco, 2006; Van Mier et al., 2019). This builds on research which argues that differing attitudes and gender stereotypes in math education should be refuted (Devine et al., 2012). This is especially necessary in light of recent findings suggesting that female teachers and mothers underestimate girls’ mathematics performance relative to boys’ (McCoy et al., 2021), likely due to the societal stereotypes mentioned above. Girls were, however, observed to display higher achievement in Literacy than their male counterparts. The outcomes found here for both subjects were similar to the longitudinal findings of the PISA study (Brown & Alexandersen, 2020; Guiso et al., 2008) whereby gender gaps for mathematics were particularly small in countries with higher levels of gender equality and girls out‐performed boys in verbal and reading tasks across the board. Girls tend to have a more favourable attitude towards educational reading than boys (Di Tommaso et al., 2021; Logan & Johnston, 2009). Our findings support this view and perhaps emphasize the importance of fostering positive attitudes towards both numeracy and literacy attainment even in spite of victimization.

The results of this study highlight the need for interventions to address the psychological impact that bullying has on the victim (e.g., Berry & Hunt, 2009). Very often anti‐bullying interventions address ways in which the school can prevent bullying from occurring and while they have been shown to impact the prevalence of bullying and help victimization, they often fail to address the impact of the consequences of bullying for the victim (Gaffney et al., 2019; Huitsing et al., 2019; Van der Ploeg et al., 2016). With this in mind, focus should fall on addressing the knock on effects of bullying if it does occur.

This study had a number of strengths. Data were used from three waves of the National Longitudinal Study of Young People in Ireland, the sample size was over six thousand and the data were weighted to reflect the population of interest. The measures used to assess psychological difficulties (Strengths and Difficulties Questionnaire) and Mathematic and Literacy attainment scores (Junior Certificate examination & Drumcondra Reasoning Test) are widely used and psychometrically sound (McNamara et al., 2018; Thornton et al., 2016). The study did, however, have a number of limitations. Based on the way in which bullying was measured in the Growing Up in Ireland study, it is not possible to ascertain if the child is describing school‐based bullying or bullying by another influential person in the child's life. The analysis only included three waves of data from the Growing Up in Ireland study, once additional waves of the Growing Up in Ireland data become available, future research could investigate if bullying at age 9 has an impact on final school examination scores. This study did not account for the effects that childhood resilience may have on this relationship due to the lack of objective measures of resilience in the GUI dataset. Future research should strive to include analysis of variables which might remedy the negative psychological impact of bullying on educational achievement (Healy & Sanders, 2018; Sapouna & Wolke, 2013).

This study uses longitudinal insights gathered from the Growing Up in Ireland dataset to provide evidence for the link between psychological difficulties as a result of bullying in primary education and the negative consequences on both Numeracy and Literacy attainment throughout adolescence. The presence of such results, even when controlling for numerous demographic and educational factors, place emphasis on the requirement for high quality interventions for young victims which might mitigate or restructure the negative emotional impacts of bullying (Brown & Taylor, 2008). Successful interventions may lead not only to improvements in educational attainment, but more positive psychological and socioeconomic outcomes later in life.

## AUTHOR CONTRIBUTION

**Daráine Murphy:** Conceptualization; Formal analysis; Investigation; Methodology; Project administration; Writing – original draft; Writing – review & editing. **Sophie J. Leonard:** Formal analysis; Investigation; Methodology; Project administration; Visualization; Writing – original draft; Writing – review & editing. **Laura K. Taylor:** Formal analysis; Writing – original draft; Writing – review & editing. **Flavia H. Santos:** Conceptualization; Methodology; Project administration; Supervision; Writing – original draft; Writing – review & editing.

## CONFLICT OF INTEREST

There is no conflict of interests.

## Data Availability

Authors can share scripts and analysis code only with researchers who obtain data access grantedby The Irish Social Science Data Archive.

## References

[bjep12511-bib-0001] Beran, T. N. , Hughes, G. , & Lupart, J. (2008). A model of achievement and bullying: Analyses of the Canadian national longitudinal survey of children and youth data. Educational Research, 50(1), 25–39. 10.1080/00131880801920379

[bjep12511-bib-0003] Berry, K. , & Hunt, C. J. (2009). Evaluation of an intervention program for anxious adolescent boys who are bullied at school. Journal of Adolescent Health, 45(4), 376–382. 10.1016/j.jadohealth.2009.04.023 19766942

[bjep12511-bib-0004] Blums, A. , Belsky, J. , Grimm, K. , & Chen, Z. (2017). Building links between early socioeconomic status, cognitive ability, and math and science achievement. Journal of Cognition and Development, 18(1), 16–40. 10.1080/15248372.2016.1228652

[bjep12511-bib-0005] Brimblecombe, N. , Evans‐Lacko, S. , Knapp, M. , King, D. , Takizawa, R. , Maughan, B. , & Arseneault, L. (2018). Long term economic impact associated with childhood bullying victimisation. Social Science & Medicine, 208, 134–141. 10.1016/j.socscimed.2018.05.014 29803971

[bjep12511-bib-0006] Brown, G. R. , & Alexandersen, K. (2020). Gender equality and gender gaps in mathematics performance. Trends in Cognitive Sciences, 24(8), 591–593. 10.1016/j.tics.2020.06.002 32564986

[bjep12511-bib-0007] Brown, S. , & Taylor, K. (2008). Bullying, education and earnings: Evidence from the National Child Development Study. Economics of Education Review, 27(4), 387–401. 10.1016/j.econedurev.2007.03.003

[bjep12511-bib-0008] Campbell, M. L. , & Morrison, A. P. (2007). The relationship between bullying, psychotic‐like experiences and appraisals in 14–16‐year olds. Behaviour Research and Therapy, 45(7), 1579–1591. 10.1016/j.brat.2006.11.009 17229400

[bjep12511-bib-0009] Cao, Q. , Xu, X. , Xiang, H. , Yang, Y. , Peng, P. , & Xu, S. (2020). Bullying victimization and suicidal ideation among Chinese left‐behind children: Mediating effect of loneliness and moderating effect of gender. Children and Youth Services Review, 111, 104848. 10.1016/j.childyouth.2020.104848

[bjep12511-bib-0011] Cargnelutti, E. , Tomasetto, C. , & Passolunghi, M. C. (2017). How is anxiety related to math performance in young students? A longitudinal study of grade 2 to grade 3 children. Cognition and Emotion, 31(4), 755–764. 10.1080/02699931.2016.1147421 26935005

[bjep12511-bib-0012] Carreiras, M. , Monahan, P. J. , Lizarazu, M. , Duñabeitia, J. A. , & Molinaro, N. (2015). Numbers are not like words: Different pathways for literacy and numeracy. NeuroImage, 118, 79–89. 10.1016/j.neuroimage.2015.06.021 26067344

[bjep12511-bib-0013] Catone, G. , Marotta, R. , Pisano, S. , Lennox, B. , Carotenuto, M. , Gritti, A. , Pascotto, A. , & Broome, M. R. (2017). Psychotic‐like experiences in help‐seeking adolescents: Dimensional exploration and association with different forms of bullying victimization–A developmental social psychiatry perspective. International Journal of Social Psychiatry, 63(8), 752–762. 10.1177/2F0020764017733765 28990447

[bjep12511-bib-0014] Centers for Disease Control and Prevention . (2014). Bullying surveillance among school‐ aged children: Uniform definitions and recommended data elements . US Department of Health and Human Services.

[bjep12511-bib-0015] Chiu, M. M. , & Chow, B. W. Y. (2015). Classmate characteristics and student achievement in 33 countries: Classmates’ past achievement, family socioeconomic status, educational resources, and attitudes toward reading. Journal of Educational Psychology, 107(1), 152. 10.1037/a0036897

[bjep12511-bib-0016] Dantchev, S. , Hickman, M. , Heron, J. , Zammit, S. , & Wolke, D. (2019). The independent and cumulative effects of sibling and peer bullying in childhood on depression, anxiety, suicidal ideation, and self‐harm in adulthood. Frontiers in Psychiatry, 10, 651. 10.3389/fpsyt.2019.00651 31616323 PMC6768961

[bjep12511-bib-0017] Devine, A. , Fawcett, K. , Szűcs, D. , & Dowker, A. (2012). Gender differences in mathematics anxiety and the relation to mathematics performance while controlling for test anxiety. Behavioral and Brain Functions, 8(1), 1–9. 10.1186/1744-9081-8-33 22769743 PMC3414752

[bjep12511-bib-0018] Di Tommaso, M. L. , Maccagnan, A. , & Mendolia, S. (2021). Going beyond test scores: The gender gap in Italian children’s mathematical capability. Feminist Economics, 27(3), 161–187. 10.1080/13545701.2021.1908574

[bjep12511-bib-0019] Dobbs, J. , Doctoroff, G. L. , Fisher, P. H. , & Arnold, D. H. (2006). The association between preschool children's socio‐emotional functioning and their mathematical skills. Journal of Applied Developmental Psychology, 27(2), 97–108. 10.1016/j.appdev.2005.12.008

[bjep12511-bib-0020] Donoghue, C. , & Meltzer, L. J. (2018). Sleep it off: Bullying and sleep disturbances in adolescents. Journal of Adolescence, 68, 87–93. 10.1016/j.adolescence.2018.07.012 30067959

[bjep12511-bib-0021] Due, P. , Damsgaard, M. T. , Lund, R. , & Holstein, B. E. (2009). Is bullying equally harmful for rich and poor children?: A study of bullying and depression from age 15 to 27. The European Journal of Public Health, 19(5), 464–469. 10.1093/eurpub/ckp099 19587227

[bjep12511-bib-0022] Duncan, G. J. , Dowsett, C. J. , Claessens, A. , Magnuson, K. , Huston, A. C. , Klebanov, P. , Pagani, L. S. , Feinstein, L. , Engel, M. , Brooks‐Gunn, J. , Sexton, H. , Duckworth, K. , & Japel, C. (2007). School readiness and later achievement. Developmental Psychology, 43(6), 1428. 10.1037/0012-1649.43.6.1428 18020822

[bjep12511-bib-0023] Educational Research Centre . (2006). Drumcondra Primary Mathematics Test – Revised. Levels 3‐6 Administration Manual and Technical Manual . Dublin: Educational Research Centre, St Patrick’s College.

[bjep12511-bib-0024] Elgar, F. J. , Craig, W. , Boyce, W. , Morgan, A. , & Vella‐Zarb, R. (2009). Income inequality and school bullying: Multilevel study of adolescents in 37 countries. Journal of Adolescent Health, 45(4), 351–359. 10.1016/j.jadohealth.2009.04.004 19766939

[bjep12511-bib-0085] Enders, C. K. (2010). Applied missing data analysis. Guilford.

[bjep12511-bib-0025] Espejo‐Siles, R. , Zych, I. , & Llorent, V. J. (2020). Empathy, social and emotional competencies, bullying perpetration and victimization as longitudinal predictors of somatic symptoms in adolescence. Journal of Affective Disorders, 271, 145–151. 10.1016/j.jad.2020.03.071 32479310

[bjep12511-bib-0027] Frenzel, A. C. , Pekrun, R. , & Goetz, T. (2007). Girls and mathematics—A “hopeless” issue? A control‐value approach to gender differences in emotions towards mathematics. European Journal of Psychology of Education, 22(4), 497. 10.1007/BF03173468

[bjep12511-bib-0028] Fry, D. , Fang, X. , Elliott, S. , Casey, T. , Zheng, X. , Li, J. , Florian, L. , & McCluskey, G. (2018). The relationships between violence in childhood and educational outcomes: A global systematic review and meta‐analysis. Child Abuse & Neglect, 75, 6–28. 10.1016/j.chiabu.2017.06.021 28711191

[bjep12511-bib-0029] Gaffney, H. , Ttofi, M. M. , & Farrington, D. P. (2019). Evaluating the effectiveness of school‐bullying prevention programs: An updated meta‐analytical review. Aggression and Violent Behavior, 45, 111–133. 10.1016/j.avb.2018.07.001

[bjep12511-bib-0030] Gaffney, H. , Ttofi, M. M. , & Farrington, D. P. (2021). What works in anti‐bullying programs? Analysis of effective intervention components. Journal of School Psychology, 85, 37–56. 10.1016/j.jsp.2020.12.002 33715780

[bjep12511-bib-0031] Gavin, A. , Keane, E. , Callaghan, M. , Molcho, M. , Kelly, C. , & Nic Gabhainn, S. (2015). The Irish health behaviour in school‐aged children (HBSC) study 2014.

[bjep12511-bib-0032] Goodman, R. (2001). Psychometric properties of the strengths and difficulties Questionnaire. Journal of the American Academy of Child Adolescent Psychiatry, 40(11), 1337–1345. 10.1097/00004583-200111000-00015 11699809

[bjep12511-bib-0033] Graham, S. , Bellmore, A. , & Juvonen, J. (2003). Peer victimization in middle school: When self‐and peer views diverge. Journal of Applied School Psychology, 19(2), 117–137. 10.1300/J008v19n02_08

[bjep12511-bib-0035] Guiso, L. , Monte, F. , Sapienza, P. , & Zingales, L. (2008). Culture, gender, and math. Science, 320(5880), 1164–1165. 10.1126/science.1154094 18511674

[bjep12511-bib-0036] Hanish, L. D. , & Guerra, N. G. (2002). A longitudinal analysis of patterns of adjustment following peer victimization. Development and Psychopathology, 14(1), 69–89. 10.1017/S0954579402001049 11893095

[bjep12511-bib-0037] Hawes, D. J. , & Dadds, M. R. (2004). Australian data and psychometric properties of the strengths and difficulties Questionnaire. Australian and New Zealand Journal of Psychiatry, 38, 644–651. 10.1080/j.1440-1614.2004.01427.x 15298588

[bjep12511-bib-0038] Hawkins, J. D. , Kosterman, R. , Catalano, R. F. , Hill, K. G. , & Abbott, R. D. (2008). Effects of social development intervention in childhood 15 years later. Archives of Pediatrics & Adolescent Medicine, 162(12), 1133–1141. 10.1001/archpedi.162.12.1133 19047540 PMC2593733

[bjep12511-bib-0039] Healy, K. L. , & Sanders, M. R. (2018). Mechanisms through which supportive relationships with parents and peers mitigate victimization, depression and internalizing problems in children bullied by peers. Child Psychiatry & Human Development, 49(5), 800–813. 10.1007/s10578-018-0793-9 29473091

[bjep12511-bib-0041] Huang, L. (2020). Exploring the relationship between school bullying and academic performance: The mediating role of students’ sense of belonging at school. Educational Studies, 48(2), 216–232. 10.1080/03055698.2020.1749032

[bjep12511-bib-0042] Huitsing, G. , Lodder, G. M. , Oldenburg, B. , Schacter, H. L. , Salmivalli, C. , Juvonen, J. , & Veenstra, R. (2019). The healthy context paradox: Victims’ adjustment during an anti‐bullying intervention. Journal of Child and Family Studies, 28(9), 2499–2509. 10.1007/s10826-018-1194-1

[bjep12511-bib-0043] Jones, S. M. , Brown, J. L. , & Lawrence Aber, J. (2011). Two‐year impacts of a universal school‐based social‐emotional and literacy intervention: An experiment in translational developmental research. Child Development, 82(2), 533–554. 10.1111/j.1467-8624.2010.01560.x 21410922

[bjep12511-bib-0044] Juvonen, J. , Nishina, A. , & Graham, S. (2000). Peer harassment, psychological adjustment, and school functioning in early adolescence. Journal of Educational Psychology, 92(2), 349. 10.1037/0022-0663.92.2.349

[bjep12511-bib-0045] Kim, Y. S. , Koh, Y. J. , & Leventhal, B. (2005). School bullying and suicidal risk in Korean middle school students. Pediatrics, 115(2), 357–363. 10.1542/peds.2004-0902 15687445

[bjep12511-bib-0081] Kirkøen, B. , Engell, T. , Follestad, I. B. , Holen, S. , & Hagen, K. A. (2021). Early academic struggles among children with home‐based support from child welfare services. Children and Youth Services Review, 131, 106268.

[bjep12511-bib-0046] Lachance, J. A. , & Mazzocco, M. M. M. (2006). A longitudinal analysis of sex differences in math and spatial skills in primary school age children. Learning and Individual Differences, 16(3), 195–216. 10.1016/j.lindif.2005.12.001 20463851 PMC2867482

[bjep12511-bib-0047] Ladd, G. W. , Ettekal, I. , & Kochenderfer‐Ladd, B. (2017). Peer victimization trajectories from kindergarten through high school: Differential pathways for children’s school engagement and achievement? Journal of Educational Psychology, 109(6), 826. 10.1037/edu0000177

[bjep12511-bib-0048] Larkin, K. , & Jorgensen, R. (2016). ‘I Hate Maths: Why Do We Need to Do Maths?’ Using iPad video diaries to investigate attitudes and emotions towards mathematics in year 3 and year 6 students. International Journal of Science and Mathematics Education, 14(5), 925–944. 10.1007/s10763-015-9621-x

[bjep12511-bib-0049] Lauer, J. E. , Esposito, A. G. , & Bauer, P. J. (2018). Domain‐specific anxiety relates to children’s math and spatial performance. Developmental Psychology, 54(11), 2126. 10.1037/dev0000605 30265030 PMC6202174

[bjep12511-bib-0050] Li, L. , Chen, X. , & Li, H. (2020). Bullying victimization, school belonging, academic engagement and achievement in adolescents in rural China: A serial mediation model. Children and Youth Services Review, 113, 104946. 10.1016/j.childyouth.2020.104946

[bjep12511-bib-0051] Logan, S. , & Johnston, R. (2009). Gender differences in reading ability and attitudes: Examining where these differences lie. Journal of Research in Reading, 32(2), 199–214. 10.1111/j.1467-9817.2008.01389.x

[bjep12511-bib-0052] McCoy, S. , Byrne, D. , & O’Connor, P. (2021). Gender stereotyping in mothers’ and teachers’ perceptions of boys’ and girls’ mathematics performance in Ireland. Oxford Review of Education, 48(3), 341–363. 10.1080/03054985.2021.1987208

[bjep12511-bib-0053] McNamara, E. , Murphy, D. , Murray, A. , Smyth, E. , & Watson, D. (2018). Growing Up in Ireland: The lives of 17/18‐year‐olds (Child Cohort Research Report No. 7). Dublin: ESRI/TCD/DCYA.

[bjep12511-bib-0054] Murphy, D. , Williams, J. , Murray, A. , & Smyth, E. (2019). Growing Up in Ireland: *Design, instrumentation and procedures for Cohort ’98 at 17/18 years of age* . (Technical Series No. 2019‐5). Dublin: ESRI/TCD/DCYA.

[bjep12511-bib-0055] Murray, A. , McCrory, C. , Thornton, M. , Williams, J. , Quail, A. , Swords, L. , Doyle, E. , & Harris, E. (2010). Growing up in Ireland: Design, instrumentation and procedures for the child cohort. Department of Health and Children.

[bjep12511-bib-0084] Muthén, L. K. , & Muthén, B. O. (2004). Mplus: The comprehensive modeling program for applied researchers: User’s guide. 3rd ed., Muthén & Muthén.

[bjep12511-bib-0056] Nakamoto, J. , & Schwartz, D. (2010). Is peer victimization associated with academic achievement? A meta‐analytic review. Social Development, 19(2), 221–242. 10.1111/j.1467-9507.2009.00539.x

[bjep12511-bib-0057] Nansel, T. R. , Overpeck, M. , Pilla, R. S. , Ruan, W. , Simons‐Morton, B. , & Scheidt, P. (2001). Bullying behaviors among us youth: Prevalence and association with psychosocial adjustment. JAMA, 285(16), 2094–2100. 10.1001/jama.285.16.2094 11311098 PMC2435211

[bjep12511-bib-0058] Narayanan, A. , & Betts, L. R. (2014). Bullying behaviors and victimization experiences among adolescent students: The role of resilience. The Journal of Genetic Psychology, 175(2), 134–146. 10.1080/00221325.2013.834290 24796160

[bjep12511-bib-0059] Oberle, E. , Schonert‐Reichl, K. A. , Hertzman, C. , & Zumbo, B. D. (2014). Social–emotional competencies make the grade: Predicting academic success in early adolescence. Journal of Applied Developmental Psychology, 35(3), 138–147. 10.1016/j.appdev.2014.02.004

[bjep12511-bib-0060] Olweus, D. (1993). Bullying at school: What we know and what we can do. Blackwell.

[bjep12511-bib-0061] Pellegrini, A. D. , & Bartini, M. (2001). Dominance in early adolescent boys: Affiliative and aggressive dimensions and possible functions. Merrill‐Palmer Quarterly, 47(1), 142–163. 10.1353/mpq.2001.0004

[bjep12511-bib-0062] Pitsia, V. , & Mazzone, A. (2020). The association of individual and contextual variables with bullying victimisation: A cross‐national comparison between Ireland and Lithuania. European Journal of Psychology of Education, 36(4), 1095–1115. 10.1007/s10212-020-00514-0

[bjep12511-bib-0063] Quail, A. , Williams, J. , Thornton, M. , & Murray, A. (2014). A summary guide to the wave 2 of the child cohort of growing up in Ireland. Economic and Social Research Institute.

[bjep12511-bib-0064] Ritchie, S. J. , & Bates, T. C. (2013). Enduring links from childhood mathematics and reading achievement to adult socioeconomic status. Psychological Science, 24(7), 1301–1308. 10.1177/2F0956797612466268 23640065

[bjep12511-bib-0065] Romano, E. , Babchishin, L. , Pagani, L. S. , & Kohen, D. (2010). School readiness and later achievement: Replication and extension using a nationwide Canadian survey. Developmental Psychology, 46(5), 995. 10.1037/a0018880 20822218

[bjep12511-bib-0066] Rueger, S. Y. , & Jenkins, L. N. (2014). Effects of peer victimization on psychological and academic adjustment in early adolescence. School Psychology Quarterly, 29(1), 77. 10.1037/spq0000036 24015982

[bjep12511-bib-0067] Sapouna, M. , & Wolke, D. (2013). Resilience to bullying victimization: The role of individual, family and peer characteristics. Child Abuse & Neglect, 37(11), 997–1006. 10.1016/j.chiabu.2013.05.009 23809169

[bjep12511-bib-0068] Schwartz, D. , Gorman, A. H. , Nakamoto, J. , & Toblin, R. L. (2005). Victimization in the peer group and children's academic functioning. Journal of Educational Psychology, 97(3), 425. 10.1037/0022-0663.97.3.425

[bjep12511-bib-0070] Smith, J. F. , & Skrbiš, Z. (2016). Arenas of comfort and conflict: Peer relationship events and young people's educational attainment. Journal of Youth Studies, 19(5), 646–664. 10.1080/13676261.2015.1098767

[bjep12511-bib-0083] Sosu, E. M. , & Schmidt, P. (2017). Tracking emotional and behavioral changes in childhood: Does the strength and difficulties questionnaire measure the same constructs across time?. Journal of Psychoeducational Assessment, 35(7), 643–656. 10.1177/0734282916655503

[bjep12511-bib-0071] Stankov, L. , Morony, S. , & Lee, Y. P. (2014). Confidence: the best non‐cognitive predictor of academic achievement? Educational Psychology, 34(1), 9–28. 10.1080/01443410.2013.814194

[bjep12511-bib-0072] Szczygiel, M. (2021). The relationship between math anxiety and math achievement in young children is mediated through working memory, not by number sense, and it is not direct. Contemporary Educational Psychology, 65, 101949. 10.1016/j.cedpsych.2021.101949

[bjep12511-bib-0073] Takizawa, R. , Maughan, B. , & Arseneault, L. (2014). Adult health outcomes of childhood bullying victimization: Evidence from a five‐decade longitudinal British birth cohort. American Journal of Psychiatry, 171(7), 777–784. 10.1176/appi.ajp.2014.13101401 24743774

[bjep12511-bib-0074] Thornton, M. , Williams, J. , McCrory, C. , Murray, A. , & Quail, A. (2016). Growing Up in Ireland: Design, instrumentation and procedures for the Child Cohort at Wave 2 (13 years). (Child Cohort Technical Report No.3). ESRI/TCD/DCYA.

[bjep12511-bib-0075] Tomasetto, C. , Alparone, F. R. , & Cadinu, M. (2011). Girls’ math performance under stereotype threat: The moderating role of mothers’ gender stereotypes. Developmental Psychology, 47(4), 943. 10.1037/a0024047 21744956

[bjep12511-bib-0076] Van der Ploeg, R. , Steglich, C. , & Veenstra, R. (2016). The support group approach in the Dutch KiVa anti‐bullying programme: Effects on victimisation, defending and well‐being at school. Educational Research, 58(3), 221–236. 10.1080/00131881.2016.1184949

[bjep12511-bib-0077] Van Mier, H. I. , Schleepen, T. M. J. , den Berg, V. , & Fabian, C. G. (2019). Gender differences regarding the impact of math anxiety on arithmetic performance in second and fourth graders. Frontiers in Psychology, 9, 2690. 10.3389/fpsyg.2018.02690 30713516 PMC6345718

[bjep12511-bib-0078] Watts, T. W. , Duncan, G. J. , Siegler, R. S. , & Davis‐Kean, P. E. (2014). The groove of growth: How early gains in math ability influence adolescent achievement. Society for Research on Educational Effectiveness, 43 (7), 352–360 10.3102/2F0013189X14553660 PMC4719158 26806961

[bjep12511-bib-0080] Zoccolotti, P. , Angelelli, P. , Marinelli, C. V. , & Romano, D. L. (2021). A network analysis of the relationship among reading, spelling and maths skills. Brain Sciences, 11(5), 656. 10.3390/brainsci11050656 34069961 PMC8157862

